# Tetrodotoxins in Tissues and Cells of Different Body Regions of Ribbon Worms *Kulikovia alborostrata* and *K. manchenkoi* from Spokoynaya Bay, Sea of Japan

**DOI:** 10.3390/toxins16040186

**Published:** 2024-04-10

**Authors:** Anna E. Vlasenko, Alexandra O. Pereverzeva, Peter V. Velansky, Timur Yu. Magarlamov

**Affiliations:** A.V. Zhirmunsky National Scientific Center of Marine Biology, Far Eastern Branch, Russian Academy of Sciences, 690041 Vladivostok, Russia

**Keywords:** tetrodotoxin and its analogues, HPLC–MS/MS, immunohistochemistry, Nemertea, *Kulikovia alborostrata*, *Kulikovia manchenkoi*

## Abstract

Nemerteans, or ribbon worms, possess tetrodotoxin and its analogues (TTXs), neurotoxins of bacterial origin, which they presumably use for capturing prey and self-defense. Most TTXs-containing nemertean species have low levels of these toxins and, therefore, have usually been neglected in studies of TTXs functions and accumulation. In the present study, *Kulikovia alborostrata* and *K. manchenkoi*, two closely related species, were analyzed for TTXs distribution in the body using the HPLC–MS/MS and fluorescence microscopy methods. The abundance of TTXs-positive cells was determined in the proboscis, integument, and digestive system epithelium. As a result, six TTXs-positive cell types were identified in each species; however, only four were common. Moreover, the proportions of the toxins in different body parts were estimated. According to the HPLC–MS/MS analysis, the TTXs concentrations in *K. alborostrata* varied from 0.91 ng/g in the proboscis to 5.52 ng/g in the precerebral region; in *K. manchenkoi*, the concentrations ranged from 7.47 ng/g in the proboscis to 72.32 ng/g in the posterior body region. The differences observed between the two nemerteans in the distribution of the TTXs were consistent with the differences in the localization of TTXs-positive cells. In addition, TTXs-positive glandular cell types were found in the intestine and characterized for the first time. TTXs in the new cell types were assumed to play a unique physiological role for nemerteans.

## 1. Introduction

Nemertea is a phylum of mostly marine worms comprising approximately 1300 species [1,2]. These are soft-body, predominantly carnivorous worms that use various toxins presumably to defend themselves against predators and/or for hunting [3,4,5]. Tetrodotoxin (TTX), a voltage-gated sodium channel-blocking neurotoxin, is widespread across all classes of nemerteans [6,7]. TTX and its analogues (TTXs) are exogenous toxins found in numerous marine and terrestrial taxa. These are hypothetically accumulated through the food web [8,9] and/or directly from TTX-producing bacteria inhabiting the host’s digestive system [10,11]. The concentration of TTXs varies both within and between nemertean species [6,7]. While toxin concentrations in some nemerteans reach extremely high levels, other TTX-bearing species contain trace amounts of TTXs.

In most studies on nemerteans’ TTXs, total extracts were used for analyses of toxins [6,7,9,10,12,13]. In several studies, nemertean’s body and proboscis were analyzed separately [14,15]; in some of them, body mucus was also considered [15]. Studies on intratissue distribution were fragmentary and focused on the foregut [16,17] and intestine only [18]. Later on, investigations focused mostly on the palaeonemertean cryptic species *Cephalothrix simula* [19,20,21,22] due to the extremely high toxicity of its representatives found in different areas [6,7,9,23,24], which sometimes reaches levels comparable to those recorded from pufferfishes and blue-ringed octopuses [25]. Recently, studies have been carried out that consider different aspects of *C. simula* toxicity, including the intratissue and intracellular distribution of TTXs; as a result, a contribution of TTXs to self-defense and food capture behavior is suggested [15,26]. However, the physiological role of TTXs and the importance of their accumulation in low-toxic nemertean species are not obvious and remain poorly understood. Our study aimed to identify the morphological features explaining the uptake of TTXs in low-toxic nemertean species and to determine the role that toxins play in their behavior.

Here, we analyzed two closely related heteronemerteans from a single clade, *Kulikovia alborostrata* and *K. manchenkoi* [27]. Earlier, both species were found to have similar ranges of TTXs concentrations [7] and also similar morphological characteristics of the integument [16,28]. We hypothesized that a detailed analysis of TTXs-bearing structures in the two closely related species should reveal the common patterns of toxin accumulation and identify common and specific TTXs-bearing structures. For this, we studied the proboscis, precerebral, mouth, anterior, middle, and posterior body regions of both species using a combination of HPLC–MS/MS and fluorescence microscopy with polyclonal anti-TTX antibodies. We obtained new data concerning TTXs localization in *K. alborostrata* and compared it with TTXs distribution in *K. manchenkoi*, studied for the first time. The results are expected to extend our knowledge about TTXs-accumulating structures in nemerteans and shed light on the new unique function of TTXs in nemerteans. 

## 2. Results

### 2.1. HPLC-MS/MS

**Figure 1:**
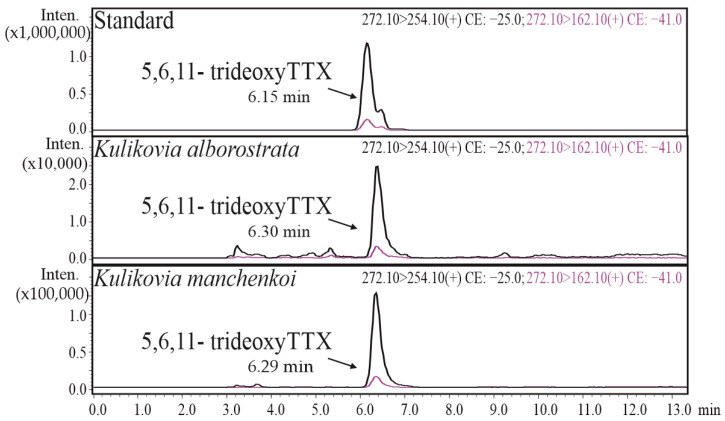
Representative high-performance liquid chromatography–tandem mass spectrometry (HPLC–MS/MS) chromatograms of a standard 5,6,11-trideoxyTTX solution and extracts of *Kulikovia alborostrata* and *K. manchenkoi*. The black and red curves represent two different mass transitions (described in each chromatogram).

The study of TTX and its analogues in different body regions (Figure 1) of *K. alborostrata* and *K. manchenkoi* showed 5,6,11-trideoxyTTX as the only toxin whose concentration was above the limit of quantification (LoQ). In several body regions of both species, TTX and 5-deoxyTTX were found at concentrations below the LoQ (Table 1 and Table 2; Figure S1). 

For the quantification of toxins, we used pooled samples of the same tissue regions from 25 specimens of *K. alborostrata* and 9 specimens of *K. manchenkoi* because the size of the tissues in some of the specimens was inadequate for estimating the toxin content. Thus, the mean weight of *K. alborostrata* specimens was 0.19 ± 0.13 g, and that of *K. manchenkoi* was 0.42 ± 0.32 g. The analyzed body parts constituted from 1 to 31% of the whole-body weight in the case of *K. alborostrata* and from 2 to 30% of the whole-body weight in the case of *K. manchenkoi*. The weights of the pooled samples are presented in Table 1 and Table 2. The analysis results were expressed as ng of toxin per 1 g of tissue of the respective body part (Table 1 and Table 2) to compare the contribution of the body part to the whole organism’s toxicity. 

Although the number of the pooled *K. alborostrata* samples was 2.5-fold larger than that of the pooled *K. manchenkoi* samples, the total 5,6,11-trideoxyTTX quantity in the *K. alborostrata* samples was 10-fold lower than in *K. manchenkoi*: 9.34 vs. 108.21 ng, respectively (Table 1 and Table 2). 

### 2.2. Morphological and Immunohistochemical Studies

**Figure 2:**
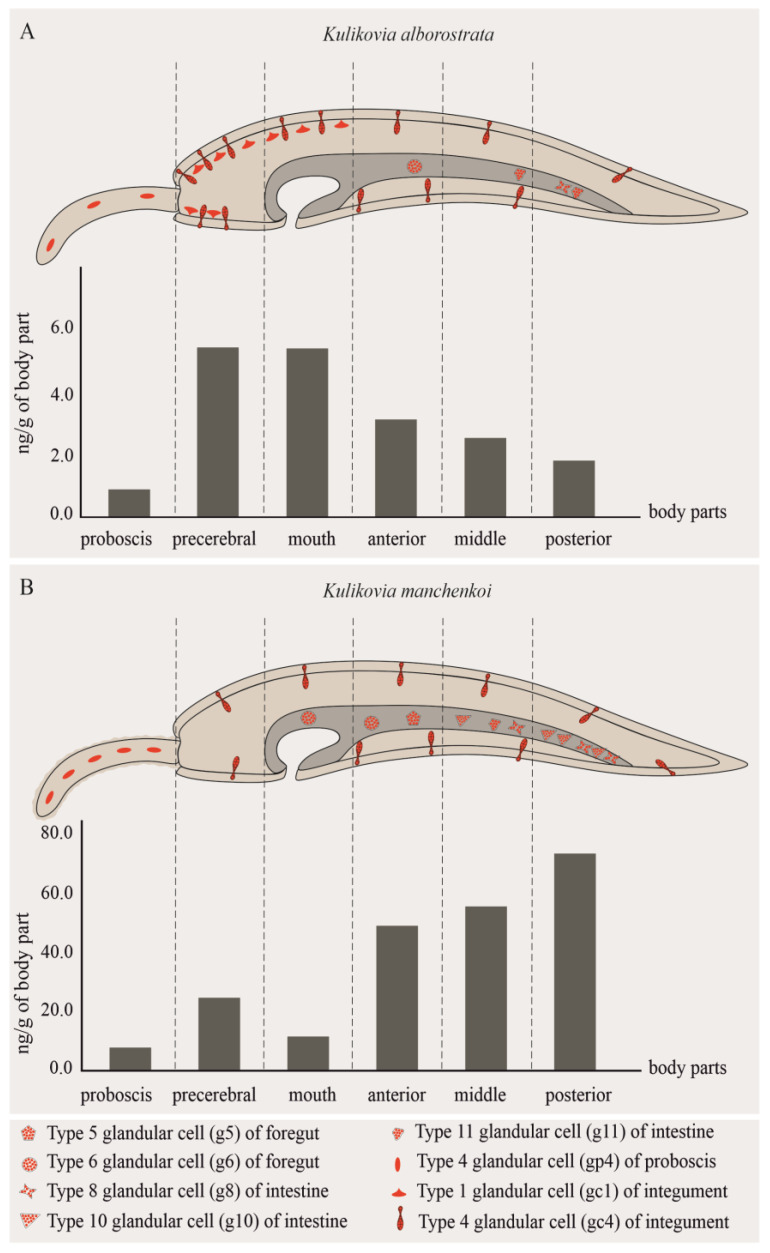
The intrabody distribution of 5,6,11-trideoxyTTX in *Kulikovia alborostrata* (**A**) and *K. manchenkoi* (**B**) as inferred by immunohistochemical studies and HPLC-MS/MS.

The fluorescent microscopy with anti-TTX antibodies revealed TTXs-like immunoreactivity in all six regions of *K. alborostrata* and *K. manchenkoi*: the proboscis, precerebral, mouth, anterior body, middle body, and posterior body regions. Both studied nemertean species showed immunofluorescent labels in the integument, intestine epithelium, and musculature and glandular epithelium of the proboscis (Table 3, Figure 2). 

#### 2.2.1. Integument

**Figure 3:**
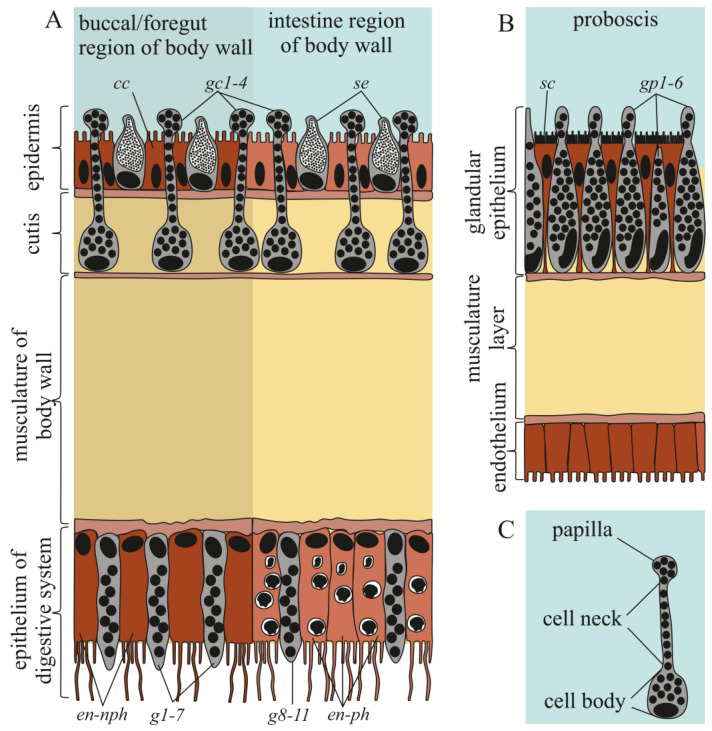
Schematic diagrams illustrating body wall (**A**), proboscis (**B**), and cutis gland cell (**C**). Letter designations: cc, ciliated cells; en-nph, non-phagocytic enterocyte; en-ph, phagocytic enterocytes; g1–11, type 1–11 glandular cells of digestive system; gc1–gc4, cutis glandular cell of types 1–4; sc, supportive cell; se, serous cell; gp1–gp5, type 1–5 glandular cells of proboscis.

**Figure 4:**
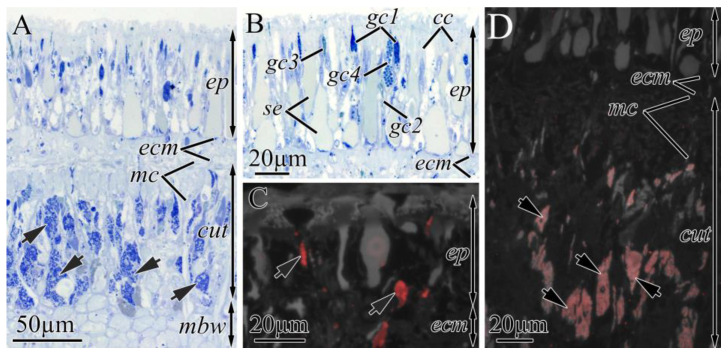
Light (**A**,**B**) and immunofluorescence (**C**,**D**) micrographs of transverse sections through integument of *Kulikovia alborostrata*. Red color indicates TTX-like immunoreactivity. (**A**) Panoramic view of integument. (**B**) Panoramic view of epidermis. (**C**) TTX-positive cytoplasmic processes (cell necks) of type 4 cutis glandular cells (arrows). (**D**) Integument of mouth region with TTX-positive cell bodies of type 1 cutis glandular cells (arrows). Letter designations: cc, ciliated cells; cut, cutis; ecm, extracellular matrix; ep, epidermis; gc1–gc4, cutis glandular cell of types 1–4; mbw, musculature of the body wall; mc, musculature of cutis; se, serous cell.

**Figure 5:**
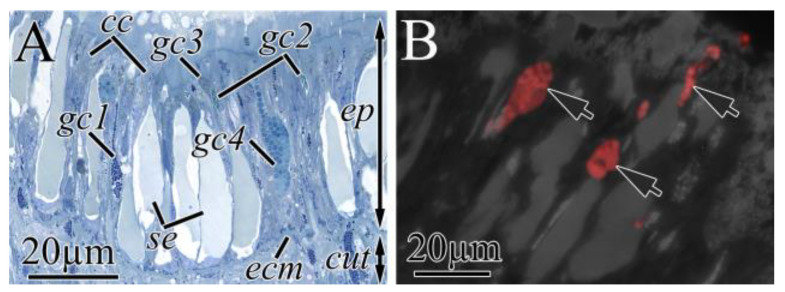
Light (**A**) and immunofluorescence (**B**) micrographs of transverse sections through integument of *Kulikovia manchenkoi*. Red color indicates TTX-like immunoreactivity. (**A**) Panoramic view of epidermis. (**B**) Distal region of epidermis with TTX-positive cytoplasmic processes (cell necks) of type 4 cutis glandular cells (arrows). Letter designations: cc, ciliated cells; cut, cutis; ecm, extracellular matrix; ep, epidermis; gc1–gc4, cutis glandular cell of types 1–4; se, serous cell.

The integument of both *K. alborostrata* and *K. manchenkoi* had a pseudostratified ciliary epithelium (epidermis) resting on the subepidermal extracellular matrix (ECM) and cutis (Figure 3 and Figure 4A). The epidermis thickness was uniform all over the nemertean’s body (40 and 54 µm in *K. alborostrata* and *K. manchenkoi*, respectively), while the cutis thickness decreased from the precerebral to the posterior regions (from 320 to 20 µm in *K. alborostrata* and from 340 to 20 µm in *K. manchenkoi*). The epidermis was mainly composed of ciliated and serous glandular cells (Figure 3A). The cutis of *K. alborostrata* and *K. manchenkoi* included cutis musculature, nerve fibers, and subepidermal (cutis) glandular cells (Figure 4A). Bodies of subepidermal glandular cells were large and irregular in shape. The apical parts of these cells formed long necks that extended through the pores of the subepidermal ECM and opened at the epidermal surface, forming papilla (Figure 3C, Figure 4B and Figure 5A). Four types of cutis glandular cells (gc1–gc4) were identified in both *K. alborostrata* and *K. manchenkoi* (Table 3). All cutis glandular cell types were evenly distributed throughout the integument (Table 3, Figure 2). 

As the immunofluorescence images show, in the integument of both nemertean species, TTXs were localized in several types of cutis glandular cells. In both species, high-intensity TTXs labeling was observed in the secretory granules of gc4; TTXs-positive secretory granules filled both bodies and necks of the cells (Figure 4C and Figure 5B). In *K. alborostrata*, weak TTXs labeling was revealed in the secretory granules of gc1; TTXs-positive secretory granules were detected in the cell bodies only (Figure 4D). TTXs-positive gc1 was found in the precerebral and mouth regions; the same cells localized in the rest of the body regions were not TTXs-positive (Figure 2).

#### 2.2.2. Digestive System

The digestive system in heteronemerteans, including *K. alborostrata* and *K. manchenkoi* studied here, consists of a mouth, a wide buccal cavity, a foregut, an intestine, and an anus [29,30,31,32]. The epithelia of the buccal cavity, the foregut, and the intestine of the middle and posterior body regions of *K. alborostrata* and *K. manchenkoi* were examined. Both the buccal cavity and the foregut epithelia were composed of non-phagocytic enterocytes and several types of glandular cells; the intestine was composed of phagocytic enterocytes and several types of glandular cells (Figure 3A). Nine types of glandular cells were found in the epithelium of the digestive system in *K. alborostrata* and *K. manchenkoi*; the cell types of both nemerteans were numbered sequentially upward (Table 3).

**Figure 6:**
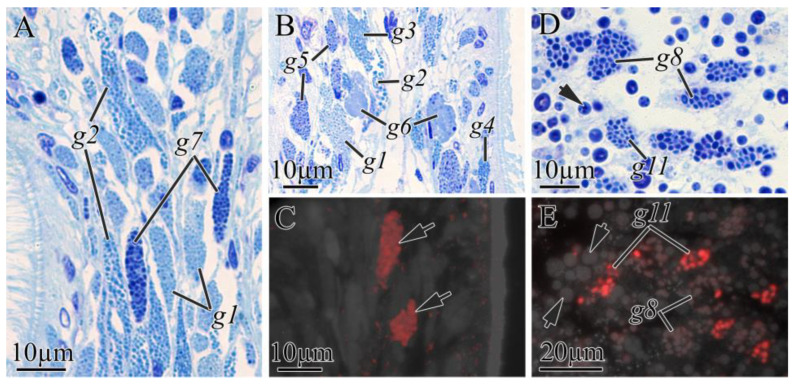
Light (**A**,**B**,**D**) and immunofluorescence (**C**,**E**) micrographs of transverse sections through digestive tract of *Kulikovia alborostrata*. Red color indicates TTX-like immunoreactivity. (**A**) Panoramic view of buccal cavity epidermis. (**B**) Panoramic view of foregut. (**C**) Distal part of foregut epithelium with TTX-positive type 6 glandular cells (arrows). (**D**) Middle region of intestinal epithelium. Arrow points to phagosome. (**E**) Intestinal epithelium with TTX-positive glandular cells. Arrows point to phagosomes. Letter designations: g1–g11, type 1–11 glandular cells of digestive system.

In the digestive system of *K. alborostrata*, there were three types of glandular cells in the buccal cavity epithelium (g1, g2, and g7) (Figure 6A), six types of glandular cells in the foregut (g1–g6) (Figure 6B), and two types of glandular cells in the intestine (g8 and g11) (Figure 6D; Table 3). Secretory granules of g6 (Figure 6C) and g11 (Figure 6E) demonstrated high-intensity TTX labeling. Secretory granules of g8 (Figure 6E) had weak TTX labeling. It should be noted that g6 was rare and located in the anterior body region only; g11 showed medium occurrence and was located in the middle and posterior body regions; and g8 was the predominant type in the middle and posterior body regions (Figure 2).

**Figure 7:**
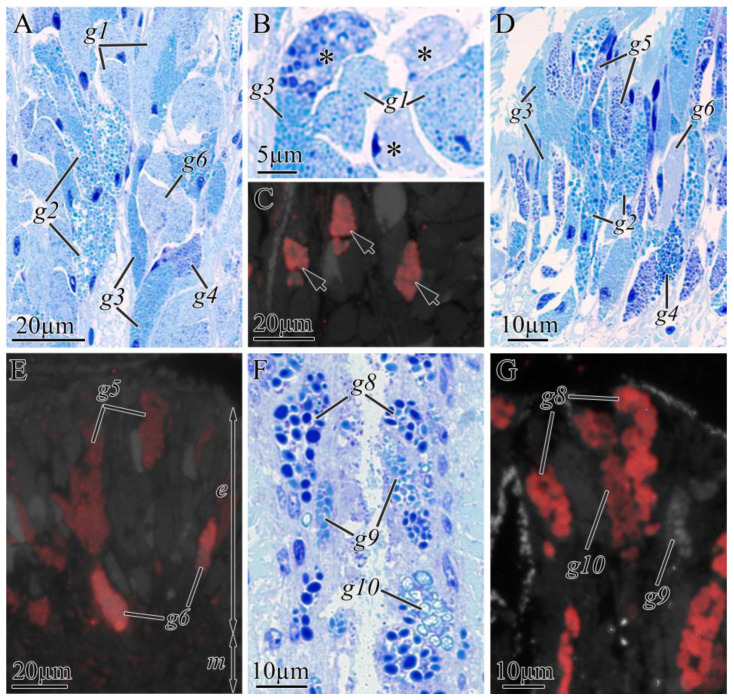
Light (**A**,**B**,**D**,**F**) and immunofluorescence (**C**,**E**,**G**) micrographs of transverse sections through digestive tract of *Kulikovia manchenkoi*. Red color indicates TTX-like immunoreactivity. (**A**) Panoramic view of buccal cavity epithelium. (**B**) Glandular cells of buccal cavity epithelium. Asterisks indicate type 6 glandular cells. (**C**) Type 6 glandular cells (arrows). (**D**) Panoramic view of foregut. (**E**) Distal region of foregut epithelium. (**F**) Panoramic view of intestine. (**G**) Distal region of intestine epithelium. Letter designations: g1–g10, type 1–10 glandular cells of digestive system.

*Kulikovia manchenkoi* had five types of glandular cells both in the buccal cavity (g1–g4 and g6) (Figure 7A) and in the foregut epithelia (g2–g6) (Figure 7D) and three types of glandular cells in the intestine (g8–g10) (Figure 7F, Table 3). In the fluorescent microscopy images, an intense TTXs-positive fluorescence was detected in the secretory granules of g6 (Figure 7C,E), g8, and g10 (Figure 7F). A medium intensity of TTXs-positive fluorescence was observed in the secretory granules of g5 (Figure 7E). g6 was rare and located in the mouth and anterior body regions; g8 was the predominant type in the middle and posterior body regions; g10 showed medium occurrence and was located in the middle and posterior body regions; and g5 was the predominant type in the anterior body region (Figure 2).

#### 2.2.3. Proboscis

**Figure 8:**
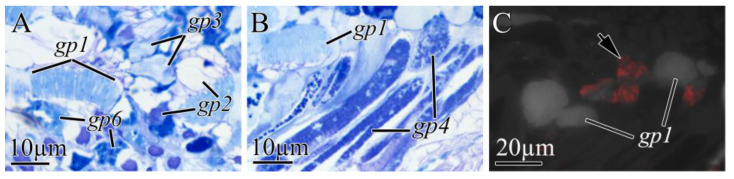
Light micrographs (**A**,**B**) and immunofluorescence (**C**) of transverse sections through proboscis of *Kulikovia alborostrata*. Red color indicates TTX-like immunoreactivity. (**A**,**B**) Panoramic view of glandular proboscis epithelium. (**C**) Middle region of glandular proboscis epithelium showing TTXs-positive glandular cell of type 4 (arrow). Letter designations: gp1–gp6, type 1–6 glandular cells of proboscis.

**Figure 9:**
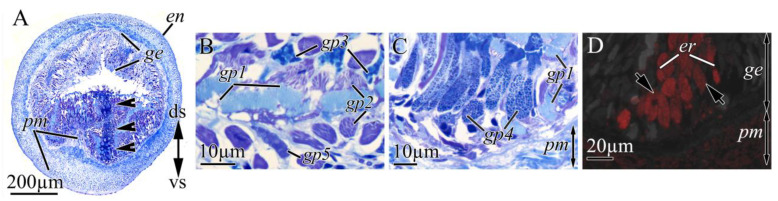
Light micrographs (**A**,**B**,**C**) and immunofluorescence (**D**) of transverse sections through proboscis of *Kulikovia manchenkoi*. Red color indicates TTX-like immunoreactivity. (**A**) Panoramic view of everted proboscis (arrows indicate epithelial ridge). (**B**,**C**) Panoramic view of glandular proboscis epithelium. (**C**) Panoramic view of glandular proboscis epithelium. (**D**) Glandular epithelium of proboscis with TTXs-positive glandular cells of type 4 (arrows). Letter designations: ds, dorsal side; en, endothelium; ge, glandular epithelium of proboscis; gp1–gp5, type 1–5 glandular cells of proboscis; pm, proboscis musculature; vs, ventral side.

The proboscises in *K. alborostrata* and *K. manchenkoi* were composed of the endothelium, three muscle layers, and the glandular epithelium (Figure 3B). In the epithelia of both *K. alborostrata* and *K. manchenkoi*, five types of glandular cells (gp1–gp6) were identified (Table 3, Figure 8A,B and Figure 9B,C). In both species, the immunohistochemical studies showed a diffuse, weak intensity of TTX labeling in the cytoplasm of endotheliocytes and proboscis musculature; also, intense TTX labeling was revealed in the secretory granules of gp4 (Figure 2, Figure 8C and Figure 9D).

## 3. Discussion

The microscopic data, as well as a chromatographic analysis, showed an uneven TTXs distribution in the bodies of both *K. alborostrata* and *K. manchenkoi* (Figure 2). Despite the close relationship of the nemertean species under study, the pattern of 5,6,11-trideoxyTTX distribution within the *K. manchenkoi* body, according to the HPLC–MS/MS analysis, was different from that in *K. alborostrata*, which was consistent with the differences in the localization of TTXs-positive cells. Thus, the high concentration of 5,6,11-trideoxyTTX in the precerebral and mouth regions of *K. alborostrata* could likely be a result of its accumulation in gc1 (Figure 4D). However, as followed from the microscopic examination, the total frequency of the occurrence of g6, g8, and g11 in *K. alborostrata* was lower than that of gc1 and gc4 in the precerebral and mouth regions, which could lead to a decrease in the 5,6,11-trideoxyTTX concentration in the anterior, middle, and posterior body regions (Table 1, Figure 2). On the contrary, gc1 was not TTXs-positive in *K. manchenkoi*, and the increase in the toxin concentration from the precerebral to posterior body regions could result from the greater abundance of TTXs-bearing glandular cells of the intestine, g8 and g10 (Figure 2 and Figure 7G). The proboscises of both studied nemertean species had similar TTXs-bearing glandular structures, gp4, whose abundances were equal, as well as similar toxin proportions (Figure 2, Figure 7C and Figure 8D; Table 1 and Table 2).

The localization of TTXs within the body in TTXs-bearing animals may indicate their physiological role [33]. Thus, TTXs were detected in the glandular cells of the proboscis and integument of some nemerteans, which gave reason to hypothesize a contribution of TTXs to prey immobilization during hunting and defense against predators [16,17,18,26]. The assumption was also confirmed by in vivo studies that demonstrated the secretion of TTXs-containing mucus from the integument [14,34]. The present study revealed the localization of the toxins in the glandular cells of the proboscis and integument epithelium of *K. alborostrata* and *K. manchenkoi* as well. However, the discovery of TTXs in the glandular cells of the intestine of both species was unexpected, and, therefore, no data concerning the secretion of the toxin in the digestive system and its role had been obtained in previous studies. 

In the proboscis, TTXs-positive cells are located in the so-called “epithelial ridge” (Figure 9D) which is formed on the proboscis’ ventral side [35]. When the proboscis is everted, the glandular epithelium appears on its external surface, and the cells of the epithelial ridge become distributed all over the extensive area of the proboscis. As was reported in a previous study, while hunting, nemertean comes in contact with its prey with the ventral side of its proboscis [36]. Therefore, a contribution of TTXs-bearing glandular cells to prey immobilization can be assumed for some of nemertean species. The first presumptive mechanism of TTXs utilization during hunting is the introduction of the toxin into the prey’s body using pseudocnidae, the structures with an internal hollow thread-like tubule (core) [37,38,39,40,41] that supposedly penetrate the prey’s integument [29,37,41]. Another possible mechanism of TTXs delivery into the prey’s body is the use of a venomous mixture of enzymes and proteinaceous pore-forming toxins produced by the proboscis that can promote TTXs distribution over tissues [42]. Thus, a comprehensive investigation should be carried out to elucidate both hypothesized TTXs delivery mechanisms.

5,6,11-trideoxyTTX was the only TTX analogue with quantified concentration in both species. Earlier, it was demonstrated that the toxic effect of 5,6,11-trideoxyTTX is minimal [43], and its contribution to nemertean’s toxicity is ambiguous. A recent behavioral study has reported that 5,6,11-trideoxyTTX elicits chemotaxis of pufferfish *Takifugu alboplumbeus* and attracts them [44]. The role of 5,6,11-trideoxyTTX in the communication of nemerteans has not been studied yet. Moreover, it should be noted that the TTXs profile of predators is not a constant and depends on the profile of TTXs sources, which may vary between different periods and localities [45]. An assumption can be made that TTXs-positive cells described here may also be involved in accumulation of toxic TTX analogues other than 5,6,11-trideoxyTTX that was dominant in the species studied here. Thus, the invariability of TTXs-accumulating structures was demonstrated for *C. simula* individuals from different localities [17,26].

The *K. alborostrata* and *K. manchenkoi* specimens examined in the present study appeared to possess common TTXs-positive gc4 in the integument that may be responsible for the secretion of the toxic mucus on the surface all over the body of both nemertean species, thus, contributing to repelling predators [14,15,34]. A microscopic examination of *K. alborostrata* also revealed gc1 whose bodies were TTXs-positive, in contrast to their ducts, which were not immunostained. Therefore, this cell type could not be involved in TTXs secretion. The function of TTXs storage could be assumed for gc1. However, further research is needed to verify this assumption. 

The presence of TTXs in the glandular cells of the intestine was demonstrated in the present study for the first time. One TTXs-positive glandular cell type was revealed in the digestive system of *K. alborostrata*, and two cell types were revealed in that of *K. manchenkoi*. The distribution of TTXs-positive glandular cells within the digestive system also varied between these species. Thus, in *K. alborostrata*, the TTXs-positive glandular cells were spread evenly within the intestine epithelium and were absent from both the foregut and the buccal cavity. In *K. manchenkoi*, TTXs-positive glandular cells were located within the digestive system, and their number/abundance increased from the buccal cavity to the posterior region of the intestine (Figure 2). Earlier, the only known TTXs-bearing cell type in the nemertean’s digestive system was enterocytes, which were assumed to uptake toxins from food [17,18,26]. The presumed way of TTXs uptake by the glandular cells of the digestive system is the toxin’s migration from enterocytes to glandular cells through the epithelium cells of the digestive system. The role of the toxins in the glandular cells of the nemertean’s digestive system, especially in the posterior intestine, seems ambiguous. Several studies have demonstrated that TTXs may help protect TTXs-bearing animals against parasitic infections, including intestinal ones [46,47]. However, the contribution of TTXs secretion into the intestine to the reduction in infections or other processes in nemerteans should be considered comprehensively.

## 4. Conclusions

The present study provides the first comprehensive overview of TTXs distribution within the body and ТТХs-accumulating structures in the low-toxic nemerteans *K. alborostrata* and *K. manchenkoi*. As a result, toxins have been detected in the glandular cells of the proboscis and integument epithelium of these nemerteans, which is consistent with the general hypothesis concerning the TTXs contribution to hunting and self-defense. However, TTXs have been discovered in the glandular cells of the intestine of both species. Nevertheless, no data concerning the secretion of the toxin in the digestive system and its function were obtained in previous studies. The newly characterized TTXs-positive glandular cells may become an incentive to study the unique unknown physiological role of TTXs in nemerteans.

## 5. Materials and Methods

### 5.1. Sample Collection and Preparation

**Figure 10:**
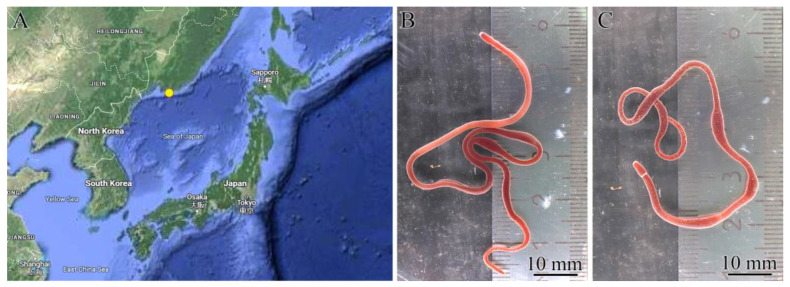
Sampling locality (**A**) of *Kulikovia alborostrata* (**B**) and *K. manchenkoi* (**C**). The images of nemerteans were taken with a reflex camera in macro mode.

**Figure 11:**
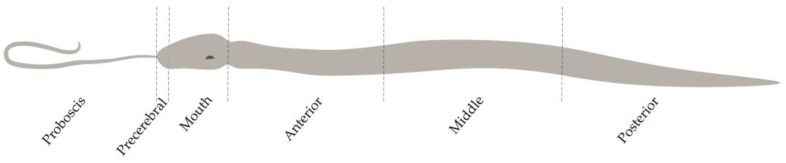
Diagram of division of *Kulikovia alborostrata* and *K. manchenkoi* for tetrodotoxin extraction and immunohistochemical and morphological studies.

*Kulikovia alborostrata* (28 specimens) and *K. manchenkoi* (12 specimens) were collected from rhizoids of the biennial brown alga *Saccharina* sp. at a depth of 0.5–2.5 m in Spokoynaya Bay, Sea of Japan (42.7090° N, 133.1809° E), in August 2023 (Figure 10). The animals were kept in containers with aerated seawater at 17 °C for three days without feeding. Then, the proboscis was cut off from each specimen, and its body was divided into five fragments: the precerebral, mouth, and anterior, middle, and posterior regions (Figure 11). To prepare a homogenate of *K. alborostrata*, these body regions of 25 specimens were pooled together; for a homogenate of *K. manchenkoi*, the same body regions of 9 specimens were pooled together. Then, both homogenates were used for the extraction of TTX and its analogues. Three specimens of each species were used for both immunohistochemical and morphological studies of all fragments.

### 5.2. Species Identification

The nemertean species were identified by sequencing the cytochrome *c* oxidase subunit I (COI) gene according to the protocol described by Chernyshev and Polyakova [48] (see Supplementary Materials). Amplification of polymerase chain reaction (PCR) was carried out using the Folmer’s primers [49]. The sequences of the COI gene were submitted to the DDBJ/ENA/GenBank databases under the accession numbers OR883925 (*K. alborostrata*) and OR883927 (*K. manchenkoi*).

### 5.3. Extraction and HPLC–MS/MS Analysis of TTX and Its Analogues

The nemertean extracts were prepared, and the analysis was carried out according to the protocol described by Vlasenko with coauthors [7]. The concentrations of the toxins were calculated following the procedure of Chen with coauthors [50]. The full protocol is provided in the Supplementary Materials.

### 5.4. Immunohistochemical Studies

For the fluorescence microscopy analysis, body fragments from the proboscis, precerebral, mouth, anterior, middle, and posterior body regions of the animals were treated according to the method described by Malykin and coauthors [26] with modifications. To confirm the specificity of the immunoreactions, the negative control was carried out according to Sato with coauthors [51]. The cross-reactivity of polyclonal anti-TTX antibodies with several TTX analogues, including 5,6,11-trideoxyTTX, was demonstrated earlier [15,52]. The full protocol is provided in the Supplementary Materials.

### 5.5. Morphological Studies

Morphological studies of nemertean body fragments from the proboscis, precerebral, mouth, anterior, middle, and posterior regions were carried out according to Pereverzeva and coauthors [16]. The full protocol is provided in the Supplementary Materials.

## Figures and Tables

**Table 1:** 5,6,11-TrideoxyTTX in extracts of *Kulikovia alborostrata*.

Body Region	Weight, g	5,6,11-TrideoxyTTX			TTX	5-DeoxyTTX
		ng/g	ng	% *		
Proboscis	0.39	0.91	0.36	3.81	−	−
Precerebral	0.04	5.52	0.20	2.19	−	−
Mouth	0.26	5.51	1.43	15.33	−	−
Anterior body	0.98	3.19	3.13	33.46	−	−
Middle body	1.10	2.59	2.84	30.45	+	−
Posterior body	0.75	1.84	1.38	14.76	−	−

*: proportion in the total amount of 5,6,11-trideoxyTTX in the body; +: <LoQ; −: not detected.

**Table 2:** 5,6,11-TrideoxyTTX in extracts of *Kulikovia manchenkoi*.

Body Region	Weight, g	5,6,11-TrideoxyTTX			TTX	5-DeoxyTTX
		ng/g	ng	% *		
Proboscis	0.30	7.47	2.24	2.07	−	−
Precerebral	0.06	24.06	1.44	1.33	−	−
Mouth	0.39	11.27	4.39	4.06	−	−
Anterior body	0.76	48.16	36.60	33.82	−	+
Middle body	0.59	54.99	32.44	29.98	−	+
Posterior body	0.43	72.32	31.10	28.74	−	+

*: proportion in the total amount of 5,6,11-trideoxyTTX in the body; +: <LoQ; −: not detected.

**Table 3:** Characteristics of glandular cells in the integument, digestive system, and proboscis of *Kulikovia alborostrata* and *K. manchenkoi*.

Cell Type	Morphological Description	Methylene Blue Staining	*Kulikovia alborostrata*			*Kulikovia manchenkoi*		
			Localization	Distribution	TTXs-Positive Immunoreactivity	Localization	Distribution	TTXs-Positive Immunoreactivity
se	single large secretory granule	light blue	epidermis	+++	−	epidermis	+++	−
gc1	small rounded secretory granules	blue-purple	cutis	+++	weak	cutis	+++	−
gc2	elongated secretory granules	blue-green	cutis	++	−	cutis	++	−
gc3	small rounded secretory granules	blue-green	cutis	+	−	cutis	+	−
gc4	large spherical secretory granules	blue	cutis	+	high	cutis	+	high
g1	small rounded secretory granules	light blue	buccal cavity, foregut	+++	−	buccal cavity	+++	−
g2	large rounded secretory granules	blue	buccal cavity, foregut	++	−	buccal cavity, foregut	++	−
g3	rounded or oval secretory granules	blue	foregut	++	−	buccal cavity, foregut	++	−
g4	rounded or oval secretory granules	purple-blue	foregut	+	−	buccal cavity, foregut	+	−
g5	rounded secretory granules	purple	foregut	+++	−	foregut	+++	medium
g6	large spherical secretory granules with heterogeneous contents	light purple	foregut	+	high	buccal cavity, foregut	+	high
g7	large rounded secretory granules	dark blue	buccal cavity	+	−	−	−	−
g8	large rounded secretory granules	blue-purple	intestine	+++	weak	intestine	+++	high
g9	rounded or oval secretory granules	light blue	−	−	−	intestine	++	−
g10	large rounded secretory granules with heterogeneous contents	light blue	−	−	−	intestine	++	high
g11	rounded secretory granules	blue-purple	intestine	++	high	−	−	−
gp1	pseudocnidae	light blue	proboscis	+++	−	proboscis	+++	−
gp2	large bacillary secretory granules	purple or light purple	proboscis	+++	−	proboscis	+++	−
gp3	large spherical secretory granules	blue or light blue	proboscis	++	−	proboscis	++	−
gp4	small rounded secretory granules	dark blue	proboscis	+	high	proboscis	++	high
gp5	large spherical secretory granules with heterogeneous content	purple	−	−	−	proboscis	++	−
gp6	rounded secretory granules with heterogeneous content	blue	proboscis	++	−	−	−	−

+++: high frequency of occurrence, ++: medium frequency of occurrence, +: low frequency of occurrence, −: not detected.

## Data Availability

All data generated and analyzed in this study are available within the article and on the Figshare repository (https://figshare.com/, accessed on 1 December 2023): https://doi.org/10.6084/m9.figshare.24709500.

## Supplementary Materials

The following supporting information can be downloaded at: https://www.mdpi.com/article/10.3390/toxins16040186/s1, Figure S1: Representative high-performance liquid chromatography-tandem mass spectrometry (HPLC–MS/MS) chromatograms of TTX and 5-deoxyTTX in the extracts of *Kulikovia alborostrata* and *K. manchenkoi*. The black and red curves represent two different mass transitions (described in each chromatogram). Figure S2: Immunofluorescence micrographs of transverse sections through the digestive tract of *K. manchenkoi*. The red color indicates TTX-like immunoreactivity. (A) Intestinal epithelium with TTX-positive glandular cells. (B) The intestinal epithelium of the control sample, treated with pre-incubated anti-TTX antibodies. Arrows indicate TTX-bearing cells with decreased staining intensity.
