# Supplementary material for: Tetrodotoxins in Tissues and Cells of Different Body Regions of Ribbon Worms Kulikovia alborostrata and K. manchenkoi from Spokoynaya Bay, Sea of Japan
Source: Toxins (Basel). 2024 Apr 10;16(4):186. doi: 10.3390/toxins16040186 (PMC11053740; doi:10.3390/toxins16040186)
Supplement: Supplementary file 1 [file toxins-16-00186-s001.zip › toxins-2877810-supplementary.pdf]

Supplementary Materials

Tetrodotoxins in Tissues and Cells of Different Body Regions of Ribbon Worms *Kulikovia alborostrata* and *K. manchenkoi* from Spokoynaya Bay, Sea of Japan

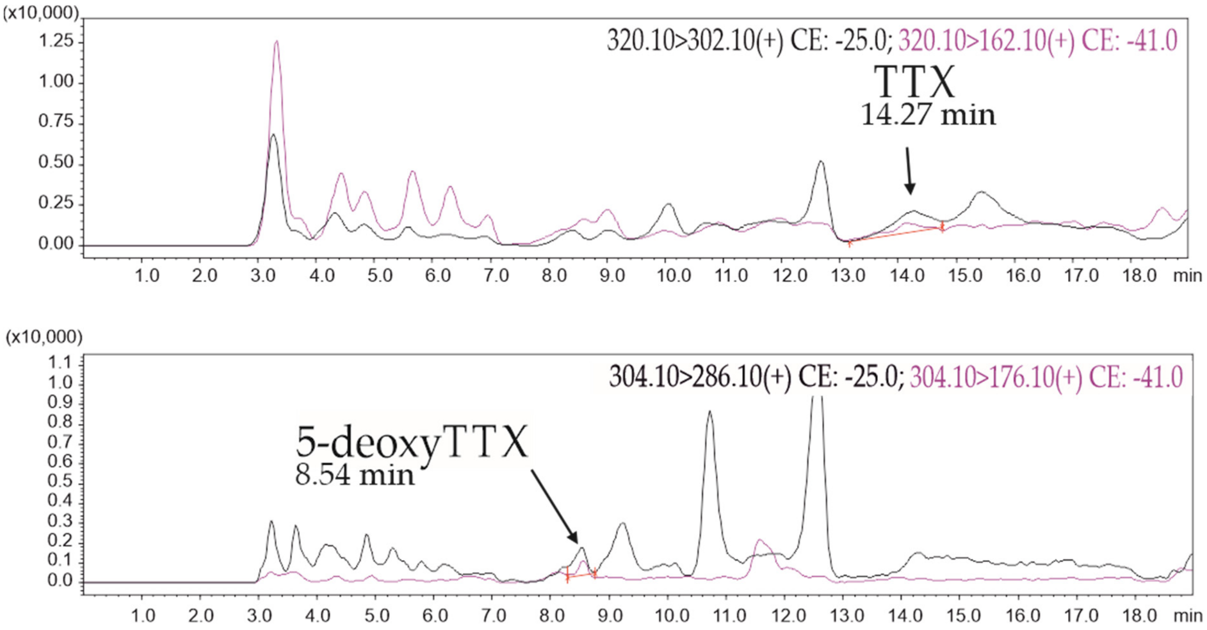

Figure S1. Representative high-performance liquid chromatography-tandem mass spectrometry (HPLC–MS/MS) chromatograms of TTX and 5-deoxyTTX in the extracts of *Kulikovia alborostrata* and *K. manchenkoi*. The black and red curves represent two different mass transitions (described in each chromatogram).

1. *Species identification*

Total genomic DNA was extracted from ethanol-fixed specimens using DNAzol (Thermo Fisher Scientific, Waltham, MA, USA) according to the manufacturer's protocol. COI gene sequences were amplified from the genomic DNA. Amplification of polymerase chain reaction (PCR) was carried out using the Folmer's primers LCO1490 GGTCAACAAATCATAAAGATATTGG and HCO2198 TAAACTTCAGGGTGACCAAAAAATCA [49]. The PCR cycling profiles were as follows: 2 min at 95°C; 40 cycles of 30 s at 95°C, 25 s at 49°C, and 1 min 30 s at 72°C; and 3 min at 72°C. Sequencing in forward and reverse directions was carried out on an ABI Prism 3500 Genetic Analyzers (Applied Biosystems, Waltham, MA, USA) under conditions recommended by the manufacturer, using a BigDye Terminator ver. 3.1 Cycle Sequencing Kit (Applied Biosystems) and the same primers as for PCR. The sequences of the COI gene were submitted to the DDBJ/ENA/GenBank databases under the accession numbers OR883925 (*K. alborostrata*) and OR883927 (*K. manchenkoi*).

2. *Extraction and HPLC–MS/MS analysis of TTX and its analogues*

Briefly, the nemertean individuals were homogenized in a 0.1% solution of acetic acid in 70% methanol (the sample/solution ratio was 1:10 v/v). The homogenates were centrifuged (14,000 × g, 10 min, 4 °C), and the supernatants were collected and evaporated. The dry precipitates were dissolved in a 0.1% aqueous solution of acetic acid, at 1 mL/g of nemertean tissue and analyzed by HPLC–MS/MS. The HPLC system

included two pairs of LC-30 pumps, a SIL-30AC autosampler, a CTO-20A thermostat, an SCL-20A system controller, and a triple quadrupole mass spectrometer LCMS-8060 (Shimadzu Europa, Duisburg, Germany), with electrostatic spray ionization (ESI). SeQuant ZIC HILIC column (150 × 2.1 mm, 5 μm) (Merck, Darmstadt, Germany) was used for separation. The toxins were detected according to the criteria of precursor MRM transition peak S/N ratio > 3, and a relative intensity of the fragment ion peak > 4%. TTX, 5,6,11-trideoxyTTX, and 5-deoxyTTX were detected using a standard solution containing these three toxins with validated structures obtained earlier from the *C.* cf. *simula*. The concentrations of the toxins were calculated following the procedure of Chen with coauthors [50], using the TTX standard as a reference peak. The method was validated using standard TTX solutions in the MRM mode. The linearity range was from 0.6 to 100 ng/mL; the recovery range from 1 to 100 ng/mL of TTX was 98.4%; the limit of quantification for TTX, 5,6,11-trideoxyTTX, and 5-deoxyTTX was determined as S/N = 10 and was 0.6 ng/mL; the LOD was determined as S/N=3 and was 0.2 ng/mL; and the relative SD was 4.5–14.6%.

3. *Immunohistochemical studies*

For the fluorescence microscopy analysis, body fragments from the proboscis, precerebral, mouth, anterior, middle, and posterior body regions of the animals were anesthetized in a 7% solution of magnesium chloride, dissected into 2–4-mm pieces, fixed in a 4% formaldehyde solution in phosphate-buffered saline (PBS; pH 7.4) for 1 h, and rinsed in PBS. The fixed material was dehydrated in a series of ethyl alcohol and acetone and embedded in LR White resin. Semithin (0.7 μm) sections were cut on a Leica UC6 ultramicrotome (Leica Microsystems) and transferred on glass slides. Some sections were stained with a methylene blue solution (Sigma-Aldrich, St. Louis, MO, USA) for morphological studies.

Sections were permeabilized for 1 h in 1% Triton-x100 in PBS, washed in PBS with 0.05% of Tween-20, incubated in a blocking solution for 1 h at 4°C (1% bovine serum albumin (BSA) in PBS), and washed in PBST. The sections were incubated with the rabbit anti-TTX antibodies (Genetex, Irvine, CA, USA) (dilution 1:25) primary antibodies for two days at 4°C, then the sections were washed in PBST, and the Alexa 647 Donkey Anti-Rabbit secondary antibodies (Invitrogen, Waltham, MA, USA) (dilution 1: 1000) were added. Secondary antibodies were incubated overnight at 4°C. Then the sections were washed with PBST, and the processed slices were embedded in Mowiol 4-88 (Sigma-Aldrich), mounted on glass slides, and analyzed on ZEISS Axio Imager A2 with a ZEISS Colibri 7 Multicolor LED Light Source (Carl Zeiss, Stuttgart, Germany). A 1% BSA solution in PBS was used as negative control, and no positive reactions were observed in any of the specimens examined. Positive control was made on the tissues of *Cephalothrix* cf. *simula*, in which TTX-positive cells were well-documented by Tanu with coauthors [17] and Malykin with coauthors [26].To confirm the specificity of the immunoreactions, the negative control was carried out according to Sato with coauthors [51]: samples were incubated in the anti-TTX antibodies pre-incubated for 2 h at RT with excessive amounts of TTX (1000 ng/ml) and processed according to the abovementioned steps; the decreasing of staining intensity was observed in all of the specimens examined (Figure S2). The cross-reactivity of polyclonal anti-TTX antibodies with several TTX analogues, including 5,6,11-trideoxyTTX, was demonstrated earlier [15,52]. Specimens were also viewed under a 647 filter to rule out aberrant autofluorescence. The obtained image series were analyzed using the ZEN 3.0 software, and the images were further processed in Adobe Photoshop 2019 (Adobe, San Jose, California, USA) to adjust the contrast and brightness and to create digital line drawings. The immunohistochemical staining intensity was calculated based on the average value of pixel within the stained field using Adobe Photoshop 2019. The intensity of proboscis muscle staining was defined as weak, the intensity of intestine g6 granules was defined as high.

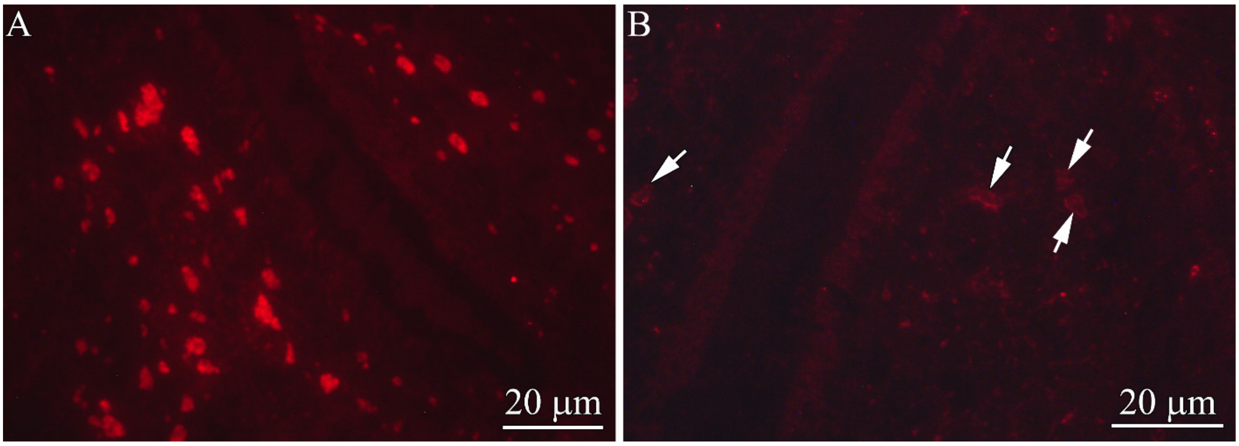

Figure S2. Immunofluorescence micrographs of transverse sections through the digestive tract of *K. manchenkoi*. The red color indicates TTX-like immunoreactivity. (**A**) Intestinal epithelium with TTX-positive glandular cells. (**B**) The intestinal epithelium of the control sample, treated with pre-incubated anti-TTX antibodies. Arrows indicate TTX-bearing cells with decreased staining intensity.

4. *Morphological studies*

The nemerteans were anesthetized in a 7% solution of magnesium chloride, dissected into 2–4-mm pieces and fixed in a 2.5% glutaraldehyde solution in a 0.2 mol L^{-1} cacodylate buffer with 0.15 mol L^{-1} sodium chloride. Postfixation was performed with 1% osmium tetroxide solution for 1 h. The fixed material was dehydrated in a series of ethyl alcohol and acetone solutions and embedded in Epon-Araldite epoxy resin (EMS, Hatfield, PA, USA). Transverse semithin sections (0.7 µm thick) were cut on a Leica UC6 ultramicrotome (Leica Microsystems, Wetzlar, Germany) and stained with methylene blue (Sigma). Observations were conducted under an Olympus IX83 microscope (Olympus, Shinjuku, Tokyo, Japan) equipped with an AxioCamHR3 camera (Carl Zeiss). Adobe Photoshop 2019 was used for image processing.
